# Cullin-5 Adaptor SPSB1 Controls NF-κB Activation Downstream of Multiple Signaling Pathways

**DOI:** 10.3389/fimmu.2019.03121

**Published:** 2020-01-21

**Authors:** Iliana Georgana, Carlos Maluquer de Motes

**Affiliations:** Department of Microbial Sciences, University of Surrey, Guildford, United Kingdom

**Keywords:** SPSB1, Cullins, NF-κB, innate immunity, inflammation, signal transduction, ubiquitin ligase, respiratory viral infection

## Abstract

The initiation of innate immune responses against pathogens relies on the activation of pattern-recognition receptors (PRRs) and corresponding intracellular signaling cascades. To avoid inappropriate or excessive activation of PRRs, these responses are tightly controlled. Cullin-RING E3 ubiquitin ligases (CRLs) have emerged as critical regulators of many cellular functions including innate immune activation and inflammation. CRLs form multiprotein complexes in which a Cullin protein acts as a scaffold and recruits specific adaptor proteins, which in turn recognize specific substrate proteins for ubiquitylation, hence providing selectivity. CRLs are divided into 5 main groups, each of which uses a specific group of adaptor proteins. Here, we systematically depleted all predicted substrate adaptors for the CRL5 family (the so-called SOCS-box proteins) and assessed the impact on the activation of the inflammatory transcription factor NF-κB. Depletion of SPSB1 resulted in a significant increase in NF-κB activation, indicating the importance of SPSB1 as an NF-κB negative regulator. In agreement, overexpression of SPSB1 suppressed NF-κB activity in a potent, dose-dependent manner in response to various agonists. Inhibition by SPSB1 was specific to NF-κB, because other transcription factors related to innate immunity and interferon (IFN) responses such as IRF-3, AP-1, and STATs remained unaffected by SPSB1. SPSB1 suppressed NF-κB activation induced via multiple pathways including Toll-like receptors and RNA and DNA sensing adaptors, and required the presence of its SOCS-box domain. To provide mechanistic insight, we examined phosphorylation and degradation of the inhibitor of κB (IκBα) and p65 translocation into the nucleus. Both remained unaffected by SPSB1, indicating that SPSB1 exerts its inhibitory activity downstream, or at the level, of the NF-κB heterodimer. In agreement with this, SPSB1 was found to co-precipitate with p65 after over-expression and at endogenous levels. Additionally, A549 cells stably expressing SPSB1 presented lower cytokine levels including type I IFN in response to cytokine stimulation and virus infection. Taken together, our results reveal novel regulatory mechanisms in innate immune signaling and identify the prominent role of SPSB1 in limiting NF-κB activation. Our work thus provides insights into inflammation and inflammatory diseases and new opportunities for the therapeutic targeting of NF-κB transcriptional activity.

## Introduction

Few transcription factors have such crucial roles in the induction of innate immune and inflammatory responses as the NF-κB family (1). NF-κB is central in the pathogenesis of multiple inflammatory disorders including those in the airway by inducing the production of pro-inflammatory cytokines such as interleukins (IL) and tumor necrosis factors (TNF). In addition, NF-κB contributes to the expression of type I interferon (IFN) in association with the IFN regulatory factors (IRF)-3/7, and the activator protein (AP)-1. Once secreted IFN triggers the production of hundreds of IFN-stimulated genes (ISG) via the Janus-associated kinase (JAK)-signal transducers and activators of transcription (STAT) signaling pathway, which confer an antiviral state to surrounding cells. NF-κB thus also impacts on the host antiviral response.

In the classical NF-κB pathway, the NF-κB p65 and p50 heterodimer is held inactive in the cytosol bound to the inhibitor of κB (IκB). Degradation of IκBα and subsequent release of NF-κB can be induced by multiple cytokine receptors and pattern-recognition receptors (PRRs) including Toll-like receptors (TLRs) that recognize viral and bacterial nucleic acids and lipids. Engagement of TNF-α with its receptor on the cell surface induces a signaling cascade that involves the TNFR-associated factor 2 (TRAF-2), whereas signaling downstream of TLRs and IL-1R employs TRAF-6. Activation of these signaling pathways induces the formation of polyubiquitin chains that act as a scaffold for the recruitment of the transforming growth factor (TGF)β-activated kinase (TAK)-1 complex via its TAK-1-binding proteins (TAB)-2/3 and the IκB kinase (IKK) complex (2, 3). Once both complexes are recruited, TAK-1 catalyzes the phosphorylation and activation of the IKK catalytic subunits (IKK-α and IKK-β), which phosphorylate IκB (4). Phosphorylated IκBα is recognized by a Cullin-RING ubiquitin ligase (CRL) complex containing Cullin-1 and the F-box protein β-transducin repeat containing protein (β-TrCP), also known as FBXW11 (5, 6), that mediates its ubiquitylation and proteasome-dependent degradation (7). The essential role of the β-TrCP-containing CRL1 complex in NF-κB activation is highlighted by the long list of viruses that antagonize its function including poxviruses (8, 9), rotaviruses (10), and the human immunodeficiency virus (11).

Nuclear p65 associates with transcriptional activators such as p300/CBP and general transcription machinery and drives the expression of genes containing NF-κB responsive elements. A plethora of post-translational modifications (PTM) affecting p65 (i.e., phosphorylation, acetylation, methylation, ubiquitylation, etc.) modulate the potency of this response and its selectivity toward specific NF-κB-dependent genes (12). These modifications are critical in fine-tuning the transcriptional activity of NF-κB.

Ubiquitylation of a protein involves its covalent attachment of ubiquitin moieties that can subsequently be ubiquitylated to form chains (13). CRL are the largest family of ubiquitin ligases and are characterized by the presence of a Cullin (Cul) protein acting as a scaffold (14). There are 5 major Cul and hence CRL (CRL1-5) families, all of which share similar architecture but recruit and target different subsets of substrates. CRL substrate recognition is directed by specific substrate receptor subunits. CRL5 complexes employ substrate receptor proteins containing the suppressor of cytokine signaling (SOCS) motif (15, 16). The SOCS box in these adaptor proteins mediates interaction with the Elongin B/C proteins that associate with Cul-5, effectively forming a CRL5 complex also known as ECS (Elongin B/C-Cul5-SOCS-box protein). Analogous to F-box proteins and CRL1 complexes, the SOCS-box domain appears in combination with other protein-protein interaction domains including Ankyrin, SPRY, and Src homology domains (17, 18). These domains are responsible for the recognition of substrates via unique signatures in the primary amino acid sequence or specific PTMs, but in many cases these remain to be elucidated. The most studied CRL5 complex is that formed by the so-called SOCS proteins (SOCS1-7), which target JAKs and act as potent inhibitors of JAK-STAT signaling (19). SOCS proteins are therefore potent negative regulators of cytokine signaling, particularly IFN, suggesting that other CRL5 adaptors may evolved similar functions suppressing innate immune activation.

Here, we have performed an RNAi-based screen to assess the role of CRL5 adaptors in NF-κB signaling. Our work has identified several molecules positively and negatively regulating the pathway, in particular the SPRY and SOCS-box containing protein (SPSB)-1. Depletion of SPSB1 resulted in enhanced NF-κB activation and cytokine expression, whereas its overexpression suppressed NF-κB responses triggered by cytokines as well as viruses. Our results indicate that SPSB1 associate with p65 but does not block its translocation, which suggests that it targets released p65. SPSB1 is known to target the inducible nitric oxide synthase (iNOS) (20, 21) and has been linked with several pathways related to cancer, but no direct role for SPSB1 in controlling NF-κB activation has been reported. Our data therefore define another function for SPSB1 in innate immunity and inflammation and reveal novel regulatory mechanisms modulating NF-κB activation.

## Results

### Identification of SPSB1 as a Negative Regulator of NF-κB Signaling

**Figure 1:**
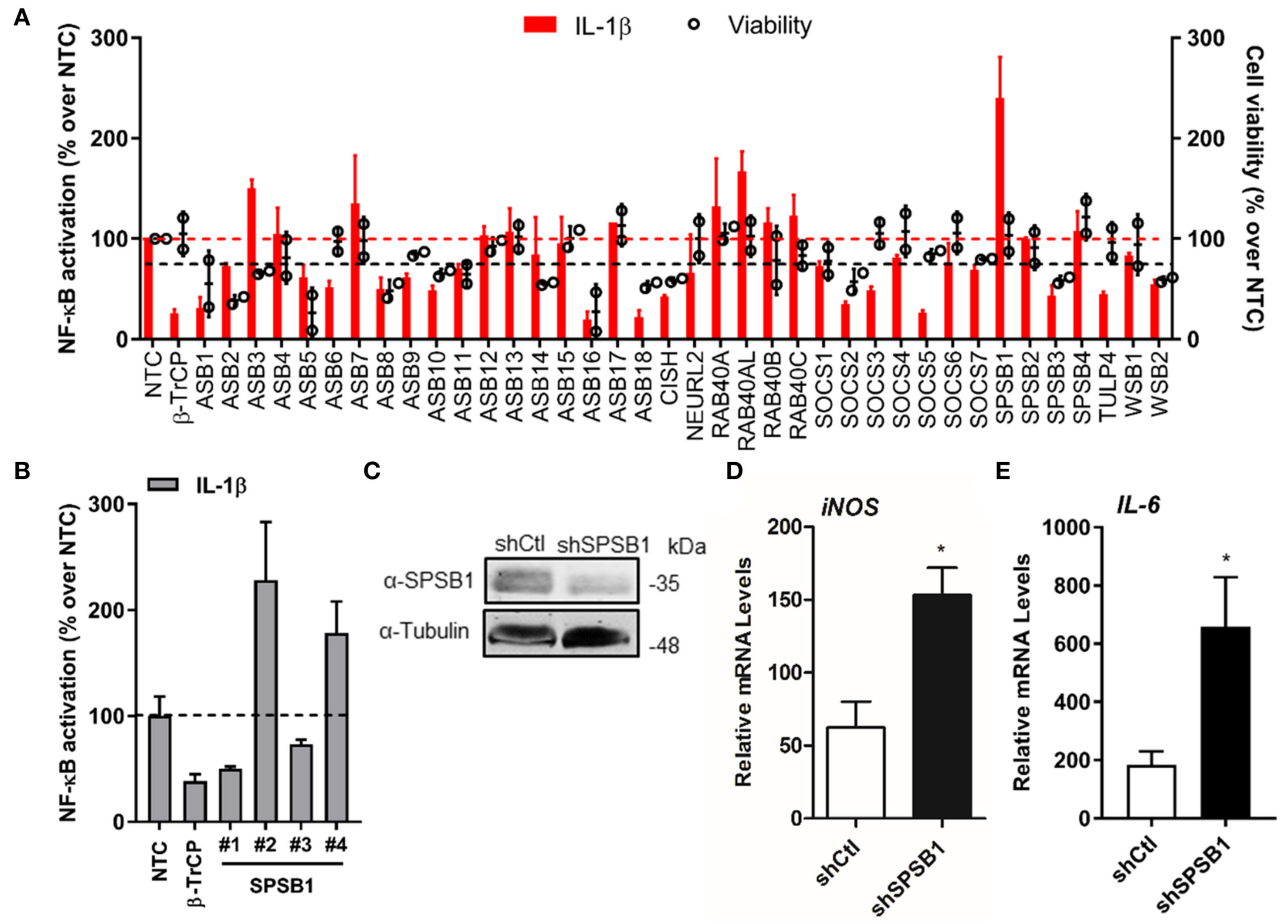
SPSB1 is a novel negative regulator of the NF-κB pathway. **(A)** IL-1β-mediated NF-κB activation (red bars, left axis) and cell viability (black circles, right axis) observed for A549-κB-Luc cells transfected with 30 nM siRNA targeting human CRL-5 adaptor proteins. Data were normalized to the non-stimulated condition and plotted as a % over NTC data. **(B)** The individual siRNA targeting SPSB1 from **(A)** were tested individually and NF-κB activation was calculated as a % over NTC data. **(C)** A549 cells were transduced with a lentiviral construct containing shRNA against SPSB1 (shSPSB1) or NTC (shCtl). Cells were lysed in RIPA buffer and subjected to immunoblotting against endogenous SPSB1 and α-tubulin. **(D,E)** shSPSB1 and shCtl cells were treated with IL-1β (25 ng/ml) for 6 h, and the mRNA levels of **(D)** *iNOS* and **(E)** *IL-6* were measured by qPCR. Means and standard deviations over the non-stimulated conditions are shown. Statistical significance was determined using an unpaired Student's *t*-test (^*^p ≤ 0.05). In all panels, data are representative of at least 2 experiments performed independently and showing similar results.

In order to identify novel members of CRLs that act as regulators of the NF-κB pathway, we set up an assay to systematically deplete CRL genes in A549 cells stably expressing the firefly luciferase gene under the control of a synthetic NF-κB promoter (22). These cells were transfected with a commercial library of siRNA designed to target all predicted human SOCS-box containing proteins (Table S1). Each gene was targeted by a pool of four individual small interfering (si)RNA duplexes. After 72 h the cells were treated with IL-1β and the luciferase activity was measured 6 h later. In a similar experiment, the viability of the cells transfected with the siRNA pools was measured. The screen was performed twice and included a non-targeting control (NTC) siRNA pool and a siRNA pool targeting β-TrCP, the CRL1 adaptor required for NF-κB activation. Each screen included triplicate replicates for each sample, and the data were normalized to the NTC (Figure 1A). As expected knock-down of β-TrCP resulted in a decrease in NF-κB activation. Amongst the 38 siRNA pools tested only SPSB1 depletion consistently resulted in a significant increase (~240%) in NF-κB activity without showing major changes in the viability of the cells (arbitrary cut-off of >75 %), suggesting that this protein has a prominent role in controlling NF-κB signaling.

To validate these initial data, we deconvolved the pool targeting SPSB1 and transfected the 4 different siRNA separately to test their effect on NF-κB activation under the same conditions used before, including NTC and β-TrCP siRNA controls. Two siRNA (#2 and #4) replicated the data observed for the pool (Figure 1B) and this represented an H-score of 0.5, a value that supported the results from the first screen (23). We then performed stable depletion of SPSB1 via short hairpin (sh)RNA transduction. Depletion of SPSB1 in the shSPSB1 cells as compared to the NTC shCtl cells was confirmed by immunoblotting (Figure 1C). These cell lines were then used to further confirm the impact of SPSB1 on NF-κB signaling. The cells were treated with IL-1β for 6 h and the mRNA levels of the cytokines *iNOS* and *IL-6* were examined by quantitative PCR. Treatment with IL-1β resulted in 63- and 190-fold increase of *iNOS* and of *IL-6* expression in the control A549 cell line, respectively. In the absence of SPSB1, this same treatment induced a significantly higher expression of both *iNOS* and *IL-6* (150 and 660 fold, respectively) and this was statistically significant (Figures 1D,E). Taken together, these data identified SPSB1 as a novel negative regulator of the NF-κB pathway, with its depletion resulting in higher expression of pro-inflammatory NF-κB-dependent genes.

### SPSB1 Inhibits NF-κB, but Not IRF-3, AP-1, or STAT Activation

**Figure 2:**
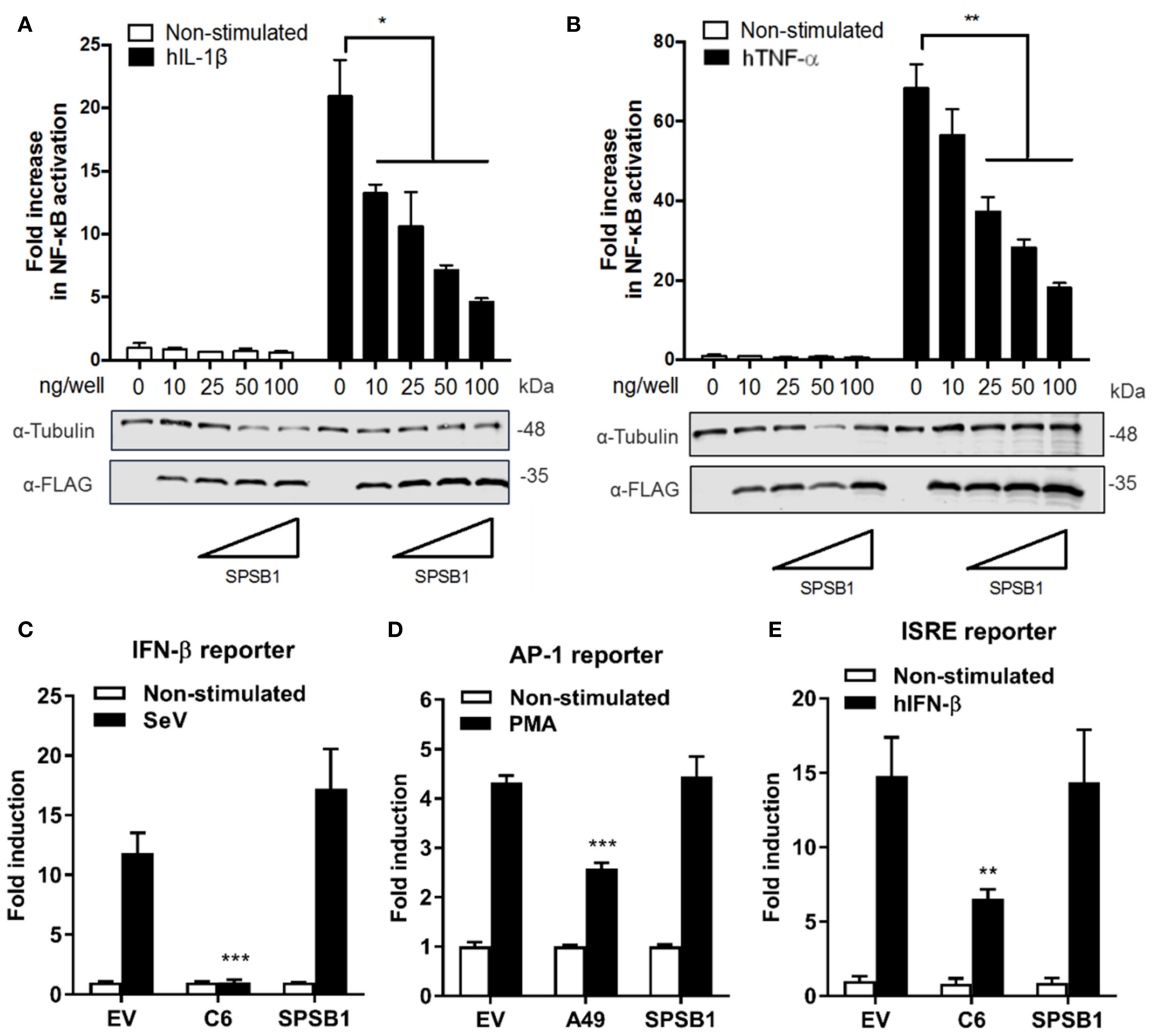
SPSB1 blocks NF-κB activation in response to IL-1β and TNF-α agonists, but it does not affect IRF-3, AP-1 or the JAK-STAT signaling pathway. **(A,B)** HEK293T cells were transfected with increasing doses of SPSB1-FLAG as indicated together with 70 ng/well of pNF-κB-LUC and 10 ng/well of pTK-Renilla reporters. After 24 h the cells were stimulated with **(A)** 20 ng/mL of IL-1β or **(B)** 50 ng/mL of TNF-α for 6 h. **(C–E)** HEK293T cells were transfected with **(C)** 70 ng/mL of pIFNβ-LUC, **(D)** 200 ng/well of pAP1-LUC, or **(D)** 70 ng/well of pISRE-LUC as well as 10 ng/well pTK-Renilla reporters, together with 25 ng of SPSB1-FLAG, the indicated positive control plasmids, or the corresponding empty vector (EV). After 24 h the cells were stimulated with **(C)** SeV for a further 24 h, **(D)** 10 ng/ml of PMA for a further 24 h, or **(E)** 50 ng/mL of IFN-β for a further 8 h. Data are representative of at least 3 independent experiments, each performed in triplicate. Means and standard deviations are shown and statistical significance was determined using an unpaired Student's *t*-test (^*^p ≤ 0.05, ^**^p ≤ 0.01, ^***^p ≤ 0.001).

To study the function of SPSB1, its sequence was cloned into a mammalian expression vector containing 3 copies of the FLAG epitope at the N terminus. SPSB1 was then tested for its ability to inhibit NF-κB activation. HEK293T cells were transfected with a reporter expressing firefly luciferase under the control of the canonical NF-κB promoter, a control reporter expressing renilla luciferase, and either SPSB1 or the corresponding empty vector (EV). After 24 h, the NF-κB pathway was stimulated with IL-1β or TNF-α for a further 6 h. The ratio of firefly and renilla luciferase activities was calculated and plotted as a fold increase over the non-stimulated EV-transfected condition. The same cell lysates were also examined by immunoblotting to determine SPSB1 expression levels. Stimulation by IL-1β or TNF-α triggered >20- and >60-fold increase, respectively, in reporter activity in EV-transfected samples. Expression of SPSB1 reduced the activation induced by IL-1β (Figure 2A) and TNF-α (Figure 2B) in a dose-response and statistically significant manner.

To assess the specificity of SPSB1 in controlling innate immune responses, the activation of the IRF, mitogen-activated protein kinase (MAPK)/AP-1, and JAK-STAT signaling pathways was examined using reporter gene assays specific for each pathway. To determine the impact of SPSB1 on the IRF-3 signaling pathway, cells were transfected and subsequently infected with Sendai virus (SeV), a strong inducer of IRF-3 (24). The infection induced a 13-fold activation in the control cells. This was blocked by the vaccinia virus (VACV) protein C6, a known inhibitor of IRF-3 signaling (25), but remained unaffected by SPSB1 (Figure 2C). Stimulation of the MAPK pathway was achieved by incubation with phorbol 12-myristate 13-acetate (PMA) for 24 h, which induced a 4-fold activation in the EV-transfected cells. This response was downregulated by the VACV protein A49 (26), but not by SPSB1 (Figure 2D). Finally, to address whether SPSB1 was able to impact signaling triggered by IFN via the JAK/STAT pathway, cells were transfected with a reporter expressing luciferase under the control of the IFN stimulating response element (ISRE) and stimulated with IFN-β. This treatment resulted in a 15-fold induction in both EV- as well as SPSB1-transfected cells, indicating that SPSB1 did not affect JAK/STAT signaling (Figure 2E). The above demonstrates that SPSB1 is not a general transcriptional modulator and specifically regulates NF-κB responses.

#### SPSB1 Inhibits NF-κB Activation Downstream of Multiple Effectors and Requires Its SOCS Domain

**Figure 3:**
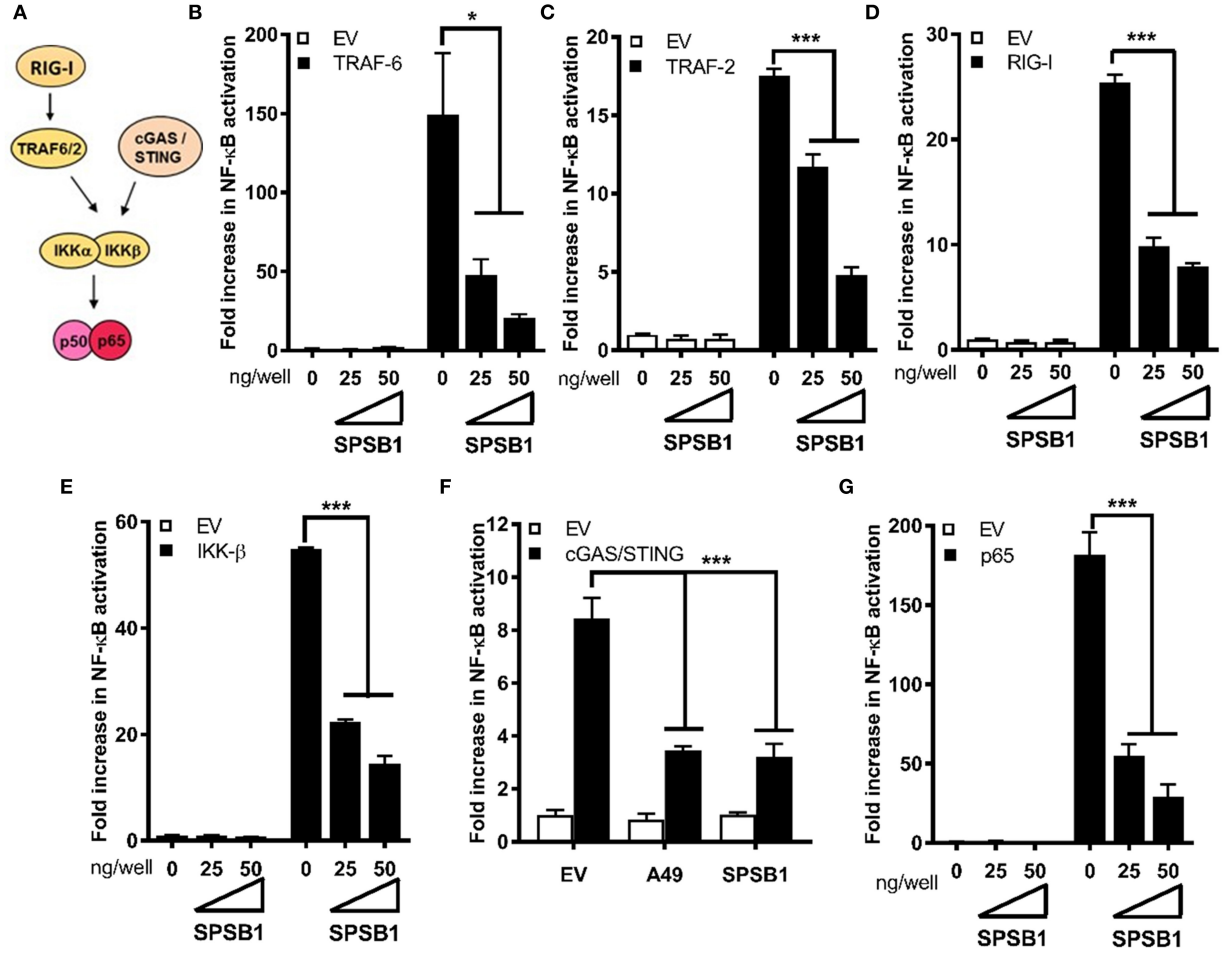
SPSB1 blocks NF-κB signaling downstream, or at the level, of the p50/p65 complex. **(A)** Schematic representation of the NF-κB key molecules and the cross-link with the cGAS/STING pathway. **(B–G)** HEK293T cells were transfected with 70 ng/well of pNF-κB-LUC and 10 ng/well of pTK-Renilla, and two doses of SPSB1-FLAG (25 and 50 ng) or an empty vector (EV). The cells were stimulated by co-transfection of **(B)** TRAF-6, **(C)** TRAF-2, **(D)** RIG-I-CARD, **(E)** IKK-β, **(F)** cGAS and STING, or **(G)** p65. VACV protein A49 was used as a control. Data are representative of at least 3 independent experiments, each performed in triplicate. Means and standard deviations are shown and statistical significance was determined using an unpaired Student's *t*-test (^*^p ≤ 0.05, ^***^p ≤ 0.001).

We then aimed to gain further insights into SPSB1 regulation of NF-κB signaling using different approaches. First, we performed luminescence-based mammalian interactome mapping (LUMIER) assays (27–29) to examine possible interactions between SPSB1 and a number of molecules operating at multiple levels downstream of the IL-1R signaling cascade (Figure 3A). SPSB1 was initially found to self-associate (Figure S1). This property was used to ensure that the fusion of SPSB1 with renilla luciferase (Rluc) did not affect its expression or folding. FLAG-SPSB1 was then co-transfected with Rluc fusions for either TRAF-6, TAK-1, TAB-2, TAB-3, IKK-α, IKK-β, IKK-γ, and β-TrCP as well as SPSB1 or an Rluc only construct. Rluc activity was measured before and after immunoprecipitation of FLAG-SPSB1 and RLuc ratios were calculated. Using this assay none of the tested NF-κB components interacted with SPSB1 (Figure S2). The second approach relied on reporter assays in which NF-κB was triggered by over-expression of signaling molecules. SPSB1 was able to suppress NF-κB activation deriving from the adaptors TRAF-6 (Figure 3B) and TRAF-2 (Figure 3C); the RNA sensor RIG-I (Figure 3D), which activates NF-κB at the level of TRAF-6; the kinase IKK-β (Figure 3E); and the DNA sensors cGAS and STING (Figure 3F), which converge on the NF-κB pathway at the level of the IKK complex. Taken together, these data indicated that SPSB1 acted downstream of these molecules. In agreement with this observation SPSB1 inhibited NF-κB activation triggered by p65 over-expression (Figure 3G).

**Figure 4:**
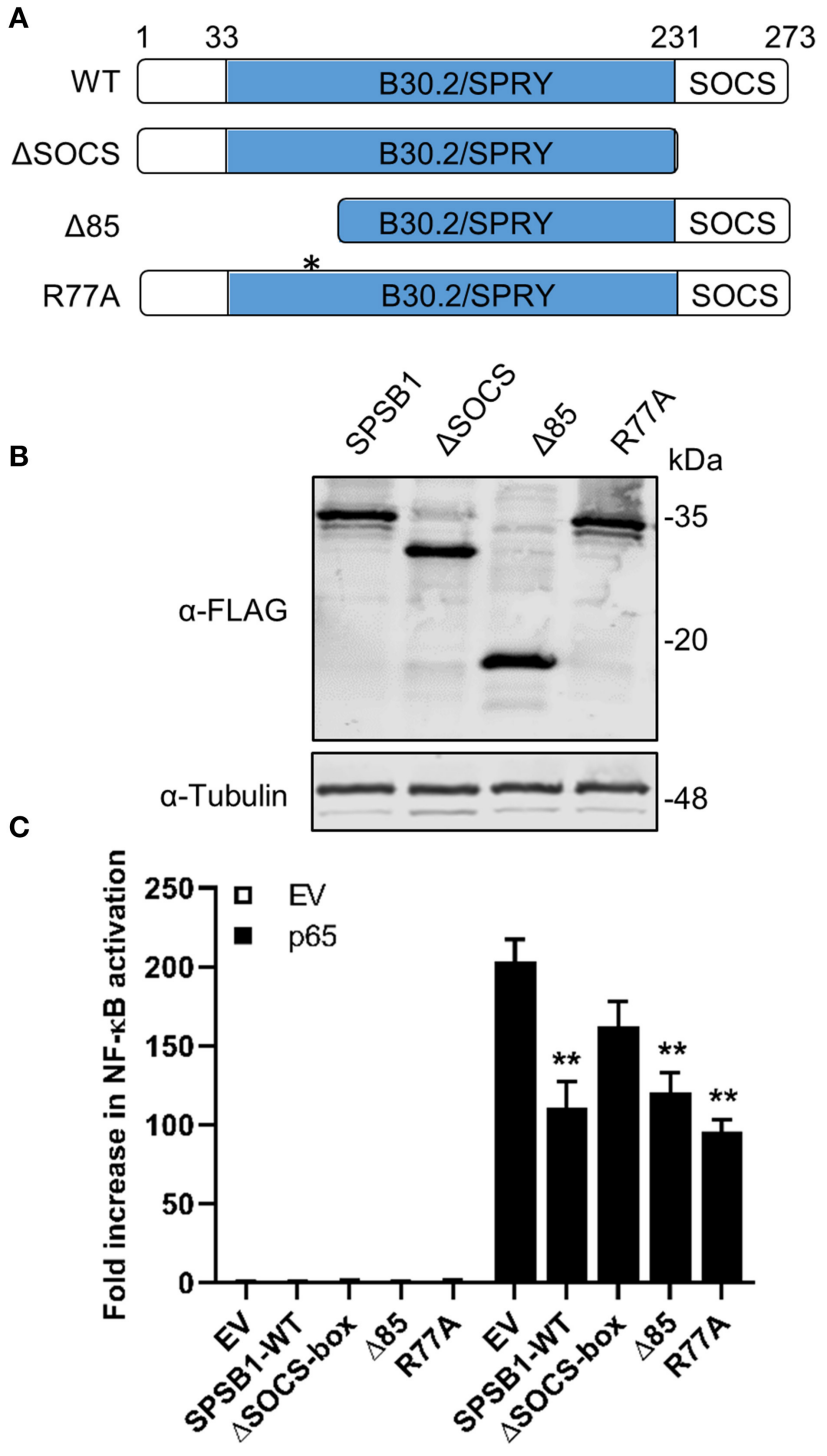
SPSB1 SOCS-box domain is needed for NF-κB inhibition. **(A)** Schematic representation of SPBS1 mutants. Asterisk indicates position for R77A mutation. **(B)** HEK293T cells were transfected with the indicated constructs and harvested in RIPA buffer 24 h after transfection. Lysates were subjected to immunoblotting against FLAG and α-tubulin. **(C)** HEK293T cells were transfected with 70 ng/well of pNF-κB-LUC and 10 ng/well of pTK-Renilla, and 25 ng/well of each SPSB1 construct. The cells were stimulated by co-transfection of p65. Data are representative of at least 3 independent experiments, each performed in triplicate. Means and standard deviations are shown and statistical significance was determined using an unpaired Student's *t*-test (^**^p ≤ 0.01).

We also created a panel of SPSB1 mutants (Figure 4A) including one lacking the entire C-terminal SOCS-box domain (ΔSOCS), one lacking the first 85 amino acids (Δ85) and one containing the point mutation R77A. These two have been described to disrupt the interaction between SPSB1 and its targets (30, 31). All these constructs expressed at similar levels (Figure 4B) and suppressed p65-induced NF-κB activation to similar extent with the exception of SPSB1-ΔSOCS, which was clearly impaired (Figure 4C). This indicated that the SOCS-box domain is needed for SPSB1 to show inhibitory activity.

#### SPSB1 Does Not Interfere With IκBα Phosphorylation or Degradation

**Figure 5:**
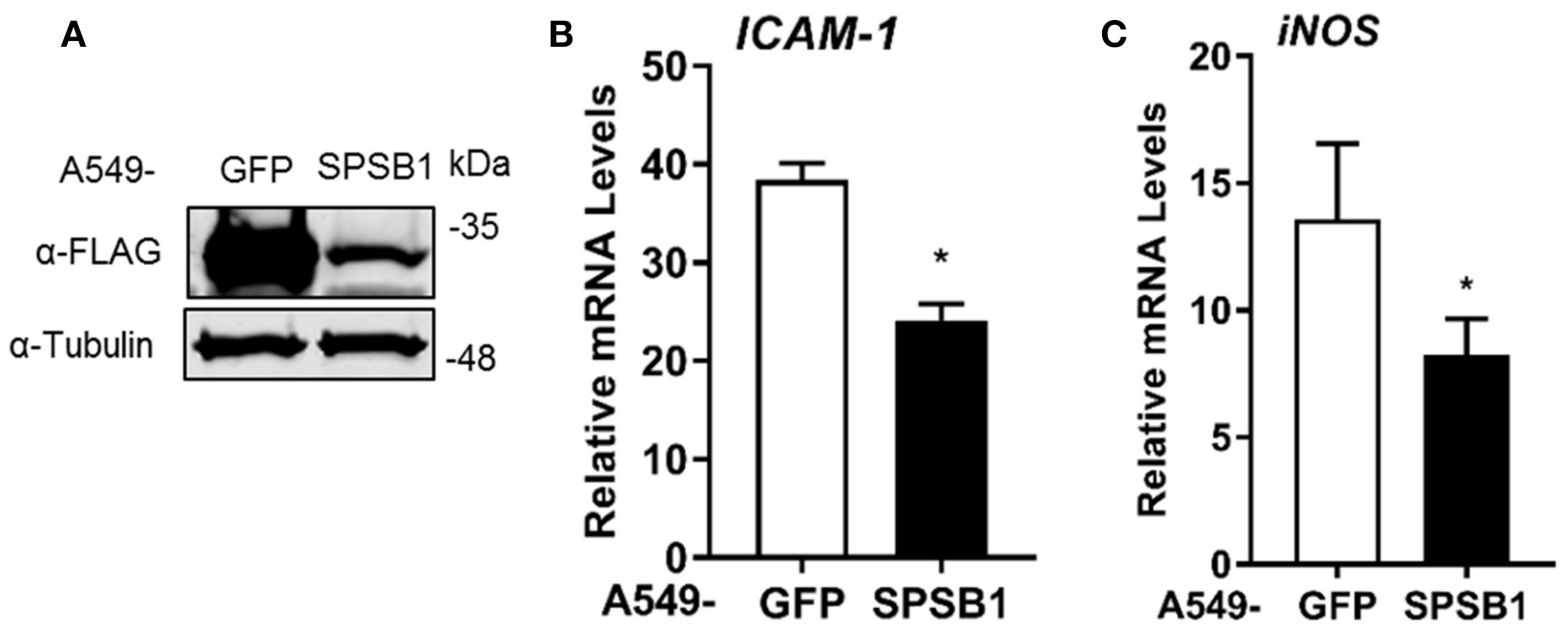
Expression levels of NF-κB-dependent genes in A549 cells stably expressing SPSB1 upon induction with IL-1β. **(A)** A549 cells were transduced with lentiviral constructs expressing FLAG-GFP or SPSB1. Cells were harvested in RIPA buffer and subjected to immunoblotting against FLAG and α-tubulin. **(B,C)** A549 cells stably expressing GFP or SPSB1 were treated with 25 ng/ml of IL-1β for 6 h. The mRNA levels of **(B)** *ICAM-1* and **(C)** *iNOS* were measured by qPCR. Data are presented as fold increase over the non-stimulated condition and representative of at least 2 independent experiments, each performed in triplicate. Means and standard deviations are shown and statistical significance was determined using an unpaired Student's *t*-test (^*^p ≤ 0.05).

If SPSB1 blocked p65-induced NF-κB activation, IκBα should be phosphorylated and degraded in the presence of SPSB1. To verify this, we first generated A549 stable cell lines expressing SPSB1 or GFP as a control protein using lentiviruses. Immunoblotting against FLAG revealed the successful transduction and expression of SPSB1 (Figure 5A). To validate that SPSB1 expression was sufficient and functional in these cells, the expression of *ICAM-1* (Figure 5B) and *iNOS* (Figure 5C), all of which contain NF-κB sites in their promoters, was assessed by quantitative PCR in response to IL-1β stimulation. The expression of all these genes was enhanced upon IL-1β treatment, but to a lower extent in cells expressing SPSB1.

**Figure 6:**
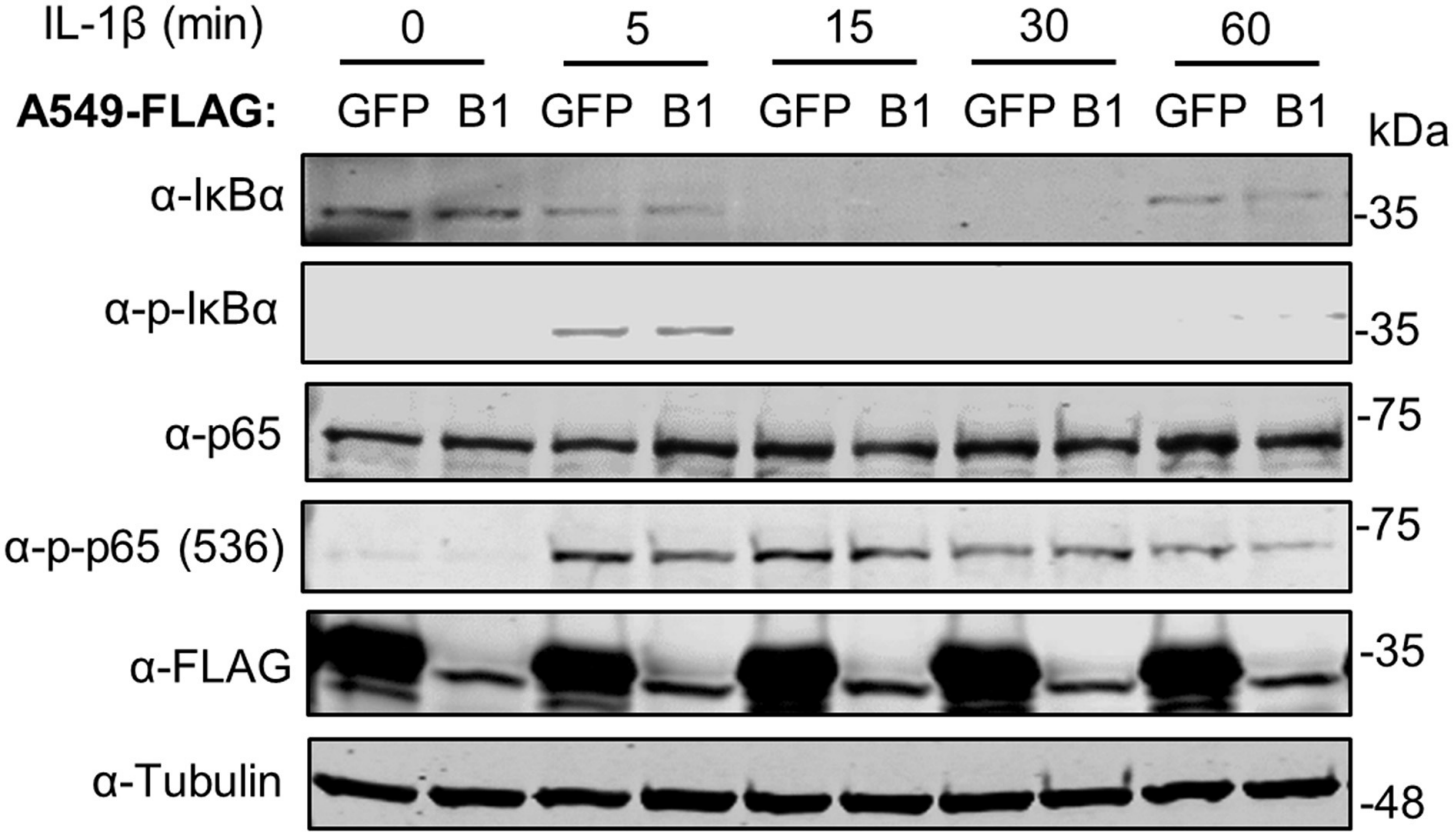
IκBα degradation in the presence of SPSB1. A549 cells stably expressing FLAG-GFP or FLAG-SPSB1 were treated with 25 ng/ml of IL-1β for the indicated length of time. Cells were harvested in RIPA buffer supplemented with protease and phosphatase inhibitors and lysates were subjected to immunoblotting against the indicated proteins or the FLAG epitope. Data are representative of 3 experiments performed independently.

We then assessed the kinetics of phosphorylation and degradation of IκBα in these cells. Exposure to IL-1β induced significant p-IκBα levels in as little as 5 min and this was concomitant with subsequent degradation of IκBα (Figure 6). The presence of SPSB1 had no effect on either the intensity or the kinetics of phosphorylation of IκBα, nor its subsequent degradation, and this was confirmed by densitometric analysis of the images (Figure S3). We also assessed the phosphorylation of Ser536 in p65, a cytoplasmic event that relates to p65 activation (32). No differences in p-p65 levels were observed between SPSB1 and its control cell line (Figure 6), and this was also confirmed by densitometry (Figure S3). In addition, the total levels of p65 remained similar in the presence of SPSB1, suggesting that this protein does not affect p65 turnover.

#### SPSB1 Does Not Interfere With p65 Translocation

**Figure 7:**
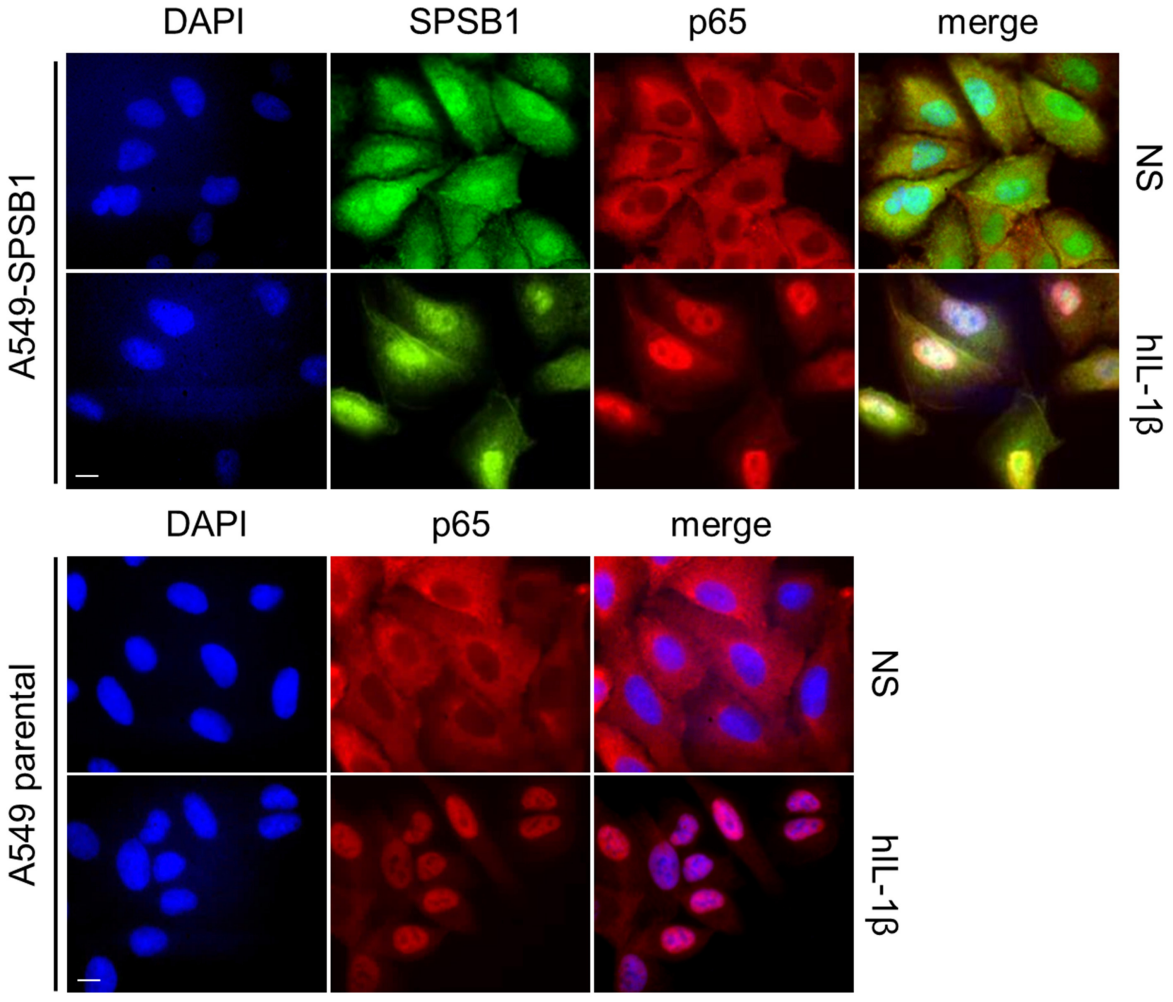
SPSB1 does not interfere with p65 translocation. A549 cells stably expressing FLAG-SPSB1 or their parental control were treated with 25 ng/mL of IL-1β for 30 min or left unstimulated (NS). The cells were then fixed and stained for FLAG (green) and p65 (red) together with DAPI (blue) to visualize cell nuclei. Merged images are also shown. Images are representative of multiple visual fields showing the same results. Bar, 10 μm.

When IκBα is phosphorylated and degraded, the NF-κB heterodimer is free to translocate into the nucleus and induce the expression of NF-κB-dependent genes. We therefore assessed whether p65 would translocate in the presence of SPSB1. Cells were challenged with IL-1β and after 30 min stained for p65 and SPSB1 (FLAG). In control unstimulated cells p65 located in the cytosol and moved to the nucleus upon stimulation (Figure 7). In SPSB1-expressing cells, p65 translocated to the nucleus to the same extent upon IL-1β exposure and no differences were observed. This indicated that SPSB1 was not able to restrict p65 translocation. Interestingly, SPSB1 showed both nuclear and cytosolic distribution in unstimulated cells, but a notable nuclear localization after stimulation, indicating that either SPSB1, or its target, alters its cellular distribution in response to NF-κB signaling.

#### SPSB1 Associates With p65

**Figure 8:**
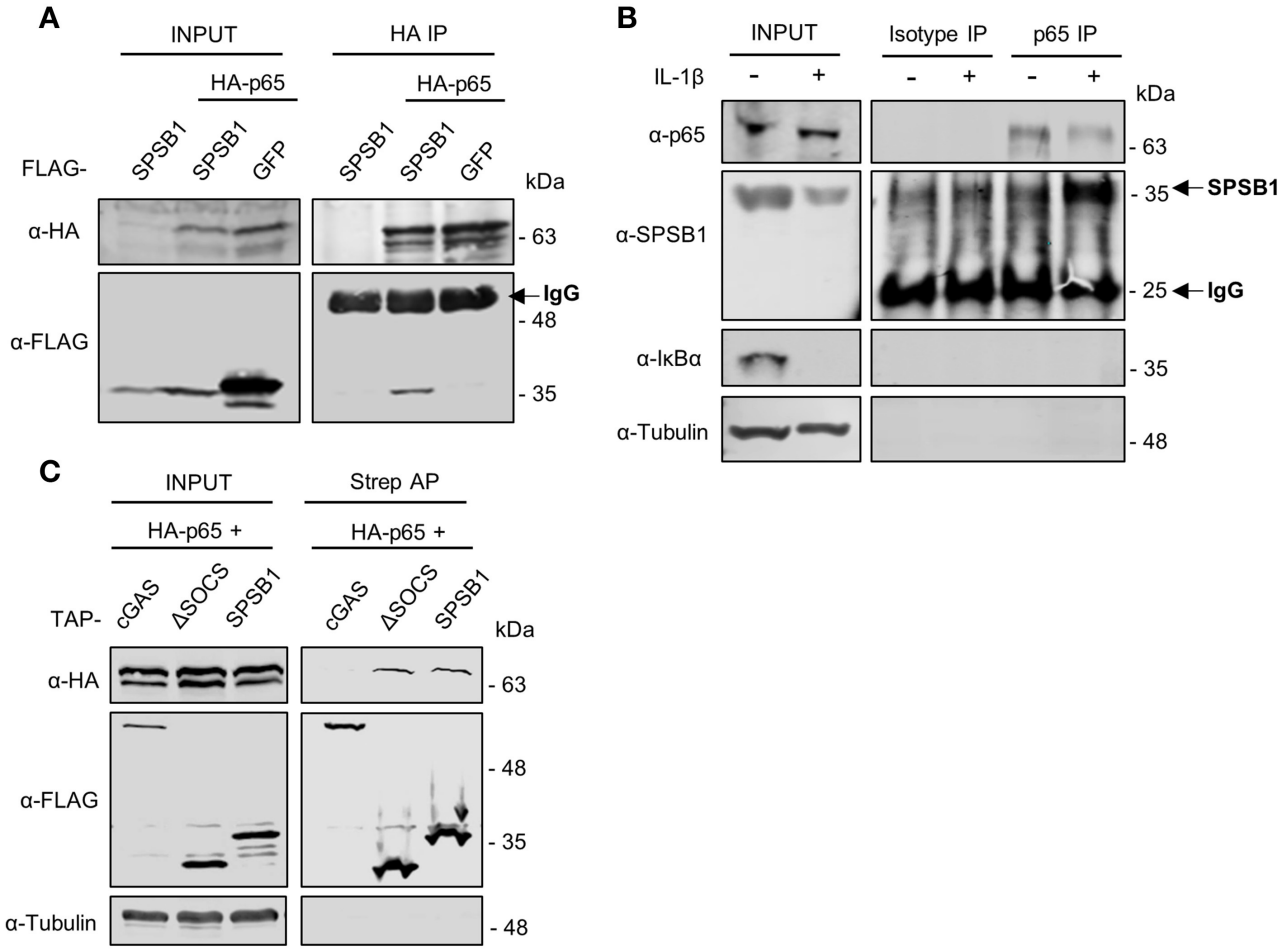
SPSB1 associates with p65. **(A)** HEK293T cells transfected with HA-p65 and FLAG-SPSB1 or GFP were lysed and subjected to HA immunoprecipitation (IP). Lysates and final IP eluates were immunoblotted (IB) against the indicated tags. IgG heavy chain is indicated with an arrow. **(B)** A549 cells were treated with 25 ng/mL of IL-1β for 30 min or left untreated and lysed in IP buffer before incubation with anti-p65 antibody or an isotype control. Both lysates and IP fractions were subjected to immunoblotting against the indicated proteins. SPSB1 and IgG light chain are indicated with arrows. **(C)** HEK293T cells transfected with HA-p65 and TAP-tagged full-length SPSB1, ΔSOCS-box or cGAS as a control were lysed and subjected to Streptavidin affinity purification (AP). Lysates and final AP eluates were immunoblotted against the indicated tags or α-tubulin.

The fact that SPSB1 shadows p65 nuclear translocation (Figure 7) and inhibits p65-induced NF-κB activation (Figure 3G) suggested an association between SPSB1 and p65. We thus immunoprecipitated HA-tagged p65 in the presence of SPSB1 or GFP as a control and observed a specific co-precipitation between SPSB1 and p65 (Figure 8A). To confirm this interaction at naturals levels, we immunoprecipitated endogenous p65 from A549 cells treated with IL-1β for 30 min or left untreated. Despite some unspecific binding in the isotype control samples we observed a significant enrichment for SPSB1 in the p65 pull-down from cells previously treated with IL-1β (Figure 8B), indicating that activation of the pathway enhances the interaction between SPSB1 and p65. This is in agreement with the prominent nuclear localization of SPSB1 after IL-1β treatment (Figure 7) and its capacity to inhibit NF-κB activation after ectopic expression of p65 (Figure 3G). Given the inability of SPSB1-ΔSOCS to suppress NF-κB activation, we also assessed the potential interaction between this mutant and p65. Full-length SPSB1, SPSB1-ΔSOCS, and cGAS as a control were expressed in HEK293T cells and subjected to affinity purification and immunoblotting (Figure 8C). Whilst no binding was observed for cGAS, both full-length and ΔSOCS interacted efficiently with p65, indicating that the SOCS-box domain is dispensable for binding to p65. Given that the SOCS-box domain is known to mediate interaction with Cul-5, these data suggest that although SPSB1-ΔSOCS interacts with p65, this is not sufficient to inhibit p65 transcriptional activity and that this requires engagement with CRL5 complexes, presumably to allow ubiquitylation. Collectively, these data revealed that SPSB1 is a potent suppressor of NF-κB responses that interacts with p65 after p65 activation in a manner that does not affect stability or translocation.

#### SPSB1 Inhibits NF-κB Activation Induced by RSV Infection

**Figure 9:**
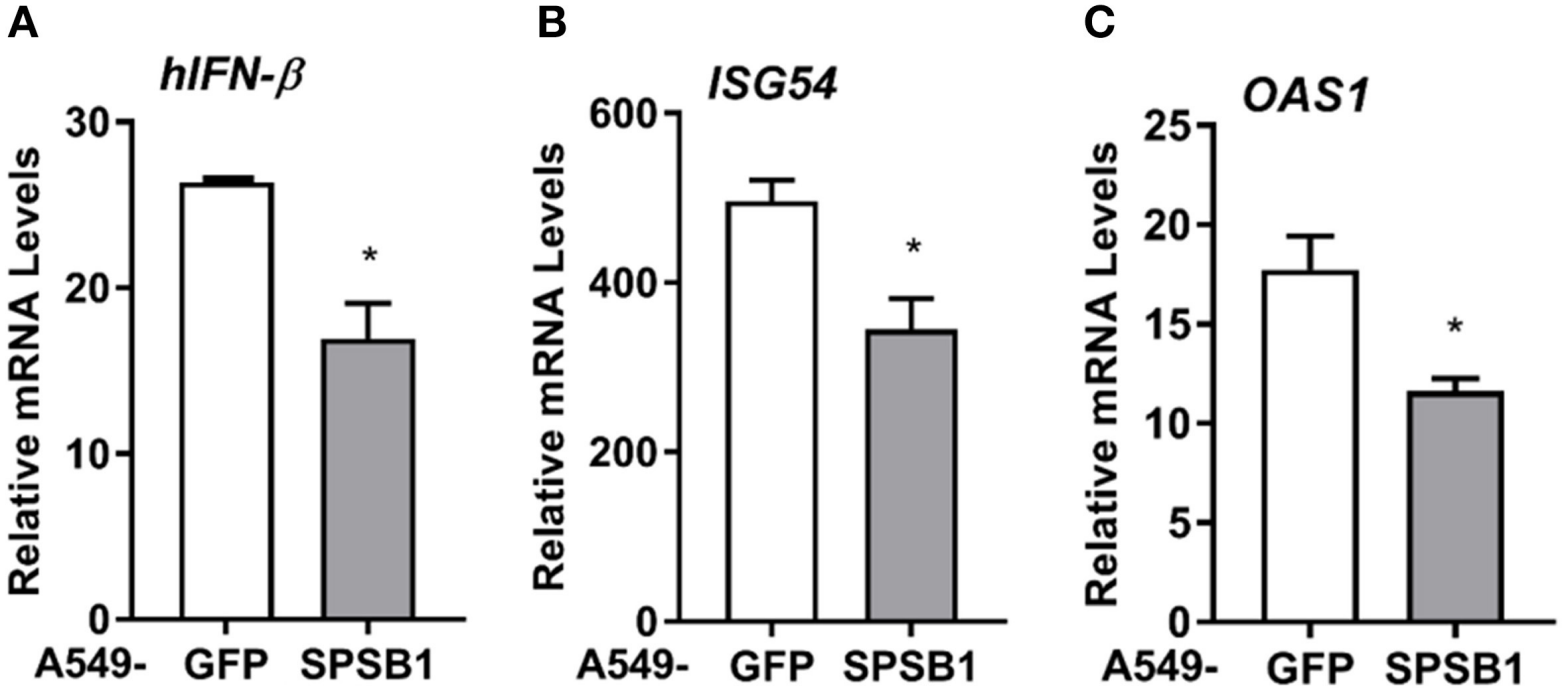
SPSB1 limits the innate immune response to RSV infection. A549 cells stably expressing GFP or SPSB1 were infected with 2 PFU/cell of RSV for 24 h. The mRNA levels of **(A)** *IFN-*β, **(B)** *ISG54*, and **(C)** *OAS1* were measured by qPCR. Data are representative of at least 3 independent experiments, each performed in triplicate. Means and standard deviations are shown and statistical significance was determined using an unpaired Student's *t*-test (^*^p ≤ 0.05).

Viruses are common inducers of NF-κB signaling. Respiratory syncytial virus (RSV) is a common respiratory pathogen and the main cause of airway inflammation in infants, and it is known to trigger NF-κB and type I IFN responses in the airway (33). To address the role of SPSB1 in controlling virus-induced responses, we infected our A549 cell lines with 2 PFU/cell of RSV and performed qPCR analysis on a number of cytokines. RSV infection triggered measurable levels of *IFN-*β in these cells and this was reduced by SPSB1 (Figure 9A). Interestingly, we also observed a significant reduction in the levels of IFN-dependent genes such as *ISG54* and *OAS1* (Figures 9B,C). Given the inability of SPSB1 to directly downregulate the JAK-STAT signaling pathway, these results indicate that SPSB1 effective suppression of IFN-β production impacted on the expression of these antiviral genes.

## Discussion

Here we have explored the role of CRL5 complexes in NF-κB activation in airway epithelial cells using an unbiased screen for SOCS-box proteins. This screen has identified SPSB1 as a novel regulator of the pathway: SPSB1 depletion resulted in enhanced NF-κB-dependent transcriptional activity (Figure 1) and this effect was reversed by SPSB1 overexpression (Figures 2, 3). In addition, our results indicate that SPSB1 controls NF-κB activation when cells were exposed to inflammatory cytokines (Figure 5) as well as viruses (Figure 9). Therefore, our work highlights SPSB1 as a novel and important participant in the signaling network that governs the production of NF-κB-dependent cytokines.

SPSB1 is the first member of the SPSB family, a group of 4 proteins (SPSB1-4) characterized by the presence of a SPRY domain and a C-terminal SOCS box domain that engages with the CRL E3 ubiquitin ligase complex (34). SPSB1, SPSB2, and SPSB4 are known to target the inducible nitric oxide synthase (iNOS) via the SPRY domain and trigger its proteasomal degradation (20, 21, 35). In addition, SPSB1 regulates multiple cancer-associated pathways via interactions with c-met (36, 37), the apoptosis-related protein Par-4 (30, 38) and the TGF-β receptor (39, 40). The specific motif that SPSB1 recognizes on its targets was suggested to be (D/E)-(I/L)-N-N-N. However, the degron recognized by SPSB1 in the TGF-β receptor has been mapped to N-I-N-H-N-T (39). The difference in sequence of the proposed motifs suggests the existence of more SPSB1 degrons than previously inventoried. Interestingly, SPSB1 has recently been shown to direct non-degradative ubiquitylation in the nucleus to regulate alternative splicing (41). Our results reveal that SPSB1 restricts the extent of NF-κB activation induced by cytokines and viruses downstream of IκBα degradation and p65 translocation and associates with p65. This indicates that SPSB1 targets p65 in the nucleus or in the cytosol in a manner that does not affect its ability to translocate. SPSB1 targeting affects the transactivation potential of p65, but not its stability. An interesting possibility is that SPSB1 mediates non-degradative ubiquitylation of p65 itself and affects its transcriptional activity perhaps by competing with other PTM known to activate p65 such as acetylation (42). Our results showing that an SPSB1 mutant that lacks the CRL5-interacting SOCS-box domain lost inhibitory capacity would support this notion, and the ability of SPSB1 to catalyze non-degradative K29 ubiquitin chains has already been described (41). Interestingly, this mutant retained the ability to interact with p65, indicating that binding to p65 is not sufficient to suppress NF-κB activation and optimal inhibition requires engagement with Cul-5, which complexes with the E2 enzyme required for ubiquitylation. Thus, SPSB1 would limit signal-induced p65 activation without triggering ubiquitin-dependent proteolysis.

An SPSB1 substrate that is of particular importance in innate immunity and inflammation is iNOS (also known as NOS2) and its catalytic product NO. iNOS is an inducible gene that is expressed at low levels in human respiratory epithelia and is upregulated in disease. Activation of NF-κB and STAT1 in response to TLR agonists and cytokines is largely responsible for the transcriptional induction of iNOS expression (43). Interestingly, SPSB1 expression is also enhanced by NF-κB and type I IFN (20). This indicates that SPSB1 expression is tightly regulated and that, according to the data presented here, represents a negative feed-back loop on NF-κB. This also implies that SPSB1 has a dual role controlling iNOS: (i) it limits iNOS expression by downregulating NF-κB activation, and (ii) it drives iNOS ubiquitylation and its proteasome-dependent destruction. SPSB1 is thus a unique molecule controlling inflammatory responses. We did not observe a role for other iNOS-modulating CLR5 complexes such as SPSB2 or SPSB4 in inhibiting NF-κB activation, although we cannot rule out that the RNAi depletion for SPSB2 and SPSB4 in our screen was not sufficiently efficient. It would therefore be interesting to assess the role of these molecules as well as its paralogue FBXO45 in the regulation of NF-κB signaling.

Our screen has also highlighted other molecules that might regulate NF-κB signaling. For instance, depletion of SOCS5 led to a substantial reduction in NF-κB activation. SOCS5 has been shown to regulate IL-4 (44) and epidermal growth factor receptor EGFR) signaling (45, 46). In addition, inhibition of EGFR/PI3K signaling by SOCS5 conferred protection against influenza infection (47). Our data suggest that SOCS5 is a critical factor required for NF-κB activation since its depletion severely impaired NF-κB reporter activation (26% upon stimulation). This may indicate that SOCS5 is necessary to activate NF-κB during viral infection and mount a protective response. We also noticed that depletion of SOCS1, a known inhibitor of NF-κB and JAK/STAT signaling (48, 49), did not result in significant changes in NF-κB activation in our screen. Further analysis of gene expression data revealed that SOCS1 is not expressed in A549 cells (50), which accounts for these results.

Excessive inflammation is central to a large number of pathologies. For instance, in the respiratory tract obstructive lung diseases such as asthma or chronic obstructive pulmonary disease (COPD) are characterized by inflammatory gene expression and the production of inflammatory mediators that enhance the recruitment of inflammatory cells (51). NF-κB is an important player in this multifactorial diseases as evidenced by the fact that the therapeutic efficacy of the main treatment for asthma—glucocorticoids—is thought to be largely caused by their ability to suppress NF-κB and AP-1 responses (52). In these diseases, NF-κB occurs largely in response to cytokines such as IL-1β and TNF-α or by infection with viruses during exacerbations (53). Mechanisms that limit this excessive inflammation are crucial to maintain homeostasis. E3 ubiquitin ligases are potent post-translational regulators with capacity to regulate inflammatory responses as recently reported for the E3 ligase TRIM29 and its critical role in regulating NEMO stability in alveolar macrophages and consequently the levels of IRF-3 and NF-κB activation (54). Here we present the novel finding that a member of the CRL5 family, SPSB1, downregulates the expression of inflammatory cytokines and other NF-κB-dependent genes in airway epithelial cells exposed to cytokines and viral infection. SPSB1 may thus have regulatory functions in chronic inflammatory disorders of the respiratory tract as well as acute virus-induced exacerbations. Our work reveals a new connection between CRL and innate immunity and may offer alternative strategies for the manipulation of NF-κB transcriptional activity in inflammatory pathologies.

## Materials and Methods

### Cells, Viruses, and Agonists

A549 and HEK293T cells were grown in Dulbecco Modified Eagle medium (DMEM, Life Technologies) supplemented with 10% heat-inactivated fetal calf serum (FCS, Biowest), 100 U/mL penicillin and 100 μg/mL Streptomycin (Pen/Strep, Invitrogen). The A549-κB-LUC cells were previously described (22). A549-SPSB1 and A549-GFP stable cell lines were grown as above with the addition of 2 μg/mL puromycin (Invitrogen). SeV strain Cantell was from Greg Towers (University College London, United Kingdom) and RSV strain A2 was from Gill Elliott (University of Surrey, United Kingdom). IL-1β, TNF-α, and IFN-β were from Peprotech. PMA was from Santa Cruz.

### RNAi Depletion Screens

siRNA sequences targeting CRL5 adaptors were purchased from Horizon Discovery and resuspended in nuclease-free water to 1 μM final concentration. A549-κB-LUC cells were reverse-transfected in triplicate replicates with 30 nM siRNA using Interferin-HTS (Polyplus) and incubated for 72 h. The cells were then stimulated with 1 ng/mL of IL-1β for 6 h and subsequently washed with ice-cold PBS and lysed in Passive Lysis Buffer (PLB; Promega). Luciferase activity was measured in a Clariostar plate reader (BMG Biotech) and data for each sample were normalized to its non-stimulated condition and plotted as mean ± SD over the NTC-transfected control. Data shown are representative of 2 independent screens showing similar results. Cells were also reverse-transfected in identical manner to determine cell viability 72 h post-transfection using CellTiter-Glo (Promega) following manufacturer's recommendations.

### shRNA Depletion and Overexpression of SPSB1 in A549 Cells

A549 cells were depleted for SPSB1 using specific short hairpin sequences expressed from the HIV-1-based shRNA expression vector HIVSiren (55). These shRNA sequences were generated using an open-access algorithm (RNAi designer, ThermoFisher) and were as follows: Top shRNA strand: 5′-GATCCACACAACCCTCGTGGGGAACGAATTCCCCACGAGGGTTGTG-3′; bottom SPSB1 strand: 5′-AATTCACAACCCTCGTGGGGAATTCGTTCCCCACGAGGGTTGTGTG-3′. Lentiviral particles were produced by transfection of HEK293T cells (seeded in 100 mm dishes) with 9 μg of pHIVSIREN shRNA, 5 μg of p8.91 packaging plasmid (56), and 3 μg of vesicular stomatitis virus-G glycoprotein expressing plasmid pMDG (Genscript) using polyethylenimine (PEI; Polysciences). Virus supernatants were harvested at 48 and 72 h post-transfection, pooled, and used to transduce A549 cells, which were subsequently selected for puromycin resistance (Invivogen, 2 μg/ml).

To generate A549 cells overexpressing SPSB1, SPSB1 was PCR amplified using primers 5′-GAAGCGGCCGCGGGTCAGAAGGTCACTGAG-3′ (fwd) and 5′-GACTCTAGATCACTGGTAGAGGAGGTAGG-3′ (rev). The PCR product was subsequently ligated into a pcDNA4/TO expression vector (Invitrogen) previously modified to express genes in frame with 3 N-terminal copies of the FLAG epitope, an N-terminal copy of the V5 epitope, or an N-terminal tandem affinity purification tag containing 2 copies of the strep tag and 1 copy of the FLAG tag as previously described (29, 57). FLAG-SPSB1 was then subcloned into a lentivirus vector carrying puromycin resistance (a gift from Greg Towers). Lentiviral particles were produced in HEK293T as above and virus supernatants were harvested at 48 and 72 h post-transfection. FLAG-SPSB1 was also used as a template to generate deletion mutants ΔSOCS (encompassing amino acids 1–231) and Δ85 (encompassing amino acids 86-273) using primers amplifying the specified regions. Mutant R77A was generated by site-directed mutagenesis using KOD hot-start DNA polymerase (Millipore).

### Quantitative PCR

RNA from confluent 6-well plates of A549 cells was purified using the Total RNA Purification Kit (Norgen Biotech). One μg of RNA was transformed into cDNA using Superscript III reverse transcriptase (Invitrogen). cDNA was diluted 1:5 in water and used as a template for real-time PCR using SYBR® Green PCR master mix (Applied Biosystems) in a LightCycler® 96 (Roche). Expression of each gene was normalized to an internal control (18S) and these values were then normalized to the shCtl or GFP control cells to yield a fold induction. Primers used for the detection of CXCL10 (58), IFNβ and 18S (59) have been described. Primers used for iNOS detection were 5′-ACAAGCCTACCCCTCCAGAT (fwd) and 5′-TCCCGTCAGTTGGTAGGTTC (rev). Data shown are representative of at least 3 independent experiments showing similar results, each performed in triplicate and plotted as mean ± SD.

### Reporter Gene Assays

HEK293T cells were seeded in 96-well plates and transfected with the indicated reporters and expression vectors using PEI as described in the figure legends. The reporter plasmids have been described previously (8). After 24 h the cells were stimulated by exposure to different agonists or by co-transfection with activating plasmids as indicated in the figure legends. Plasmids for signaling molecules have been described (57) with the exception of untagged and HA-tagged p65 that were from Geoffrey Smith (University of Cambridge, United Kingdom). TAP-tagged VACV C6 (25) and HA-tagged VACV A49 (8) have been described. After stimulation cells were washed with ice-cold PBS and lysed with PLB. Luciferase activity was measured in a Clariostar plate reader and firefly and renilla ratios were calculated for each condition. Data were normalized to mock-infected samples or samples transfected with an empty vector and presented as a fold increase. In all cases data shown are representative of at least 3 independent experiments showing similar results, each performed in triplicate and plotted as mean ± SD.

### LUMIER Assays

HEK293T cells were co-transfected with FLAG-SPSB1 and Rluc fusions for NF-κB signaling components for 24 h. Rluc fusions were from Felix Randow (Laboratory of Molecular Biology, University of Cambridge, United Kingdom) and/or have been previously described (28, 60–62). Cells were lysed in IP buffer (20 mM Tris-HCl pH7.4, 150 mM NaCl, 10 mM CaCl2, 0.1% Triton-X and 10% glycerol) supplemented with protease inhibitors (Roche) and cleared lysates were subjected to affinity purification (AP) with streptavidin beads for 6 h at 4°C. The beads were then washed 3 times with lysis buffer prior to elution with biotin (10 mg/ml) diluted in PLB. Luciferase activity was measured and data were plotted as a binding fold over Rluc-only control.

### Pull-Down Assays

HEK293T cells were seeded in 10-cm dishes and transfected with 5 μg of the indicated plasmids using PEI. After 24 h cells were lysed with IP buffer as above. Cleared lysates were incubated with HA antibody (Sigma) for 16 h at 4 °C and subsequently Protein G beads (Santa Cruz) were added for a further 2 h. For IP at endogenous levels cleared lysates from 15-cm dishes of A549 cells were incubated with p65 antibody or an isotype control and Protein G beads as above. For streptavidin affinity purification assays HEK293T cells in 10-cm dishes were transfected with 5 μg of the indicated plasmids using PEI. After 24 h cells were lysed with IP buffer as above. Cleared lysates were incubated with Streptavidin beads (Sigma) for 16 h at 4°C. The beads were then washed 3 times with IP buffer prior to incubation at 95°C for 5 min in Laemmli loading buffer to elute bound proteins. Cleared lysates and pull-down fractions were analyzed by SDS-PAGE and immunoblotting. Data shown are representative of at least 3 independent experiments showing similar results.

### SDS-PAGE and Immunoblotting

Cells were lysed in IP or RIPA buffer (50 mM Tris-HCl pH8, 150 mM NaCl, 1% NP-40, 0.5% sodium deoxycholate and 0.1% SDS) and resolved by SDS-PAGE and transferred to nitrocellulose membranes (GE Healthcare) using a Trans-Blot® semi-dry transfer unit (Bio-Rad). Membranes were blocked in 0.1% Tween PBS supplemented with 5% skimmed milk (Sigma) and subjected to immunoblotting with the following primary antibodies at the indicated dilutions: SPSB1 (Abcam, 1:1,000); FLAG (Sigma, 1:1,000); IκBα (Cell Signaling, 1:1,000); p-IκBα (S32/36) (5A5) (Cell Signaling, 1:1,000); p65 (Santa Cruz, 1:500); p-p65 (Ser536) (Santa Cruz, 1:500); V5 (BioRad, 1:5,000); α-Tubulin (Upstate Biotech, 1:10,000); HA (Sigma, 1:2,000). Primary antibodies were detected using IRDye-conjugated secondary antibodies in an Odyssey Infrared Imager (LI-COR Biosciences). Densitometric analyses were performed using Image-J.

### Immunofluorescence

Cells were seeded into 6-well plates containing sterile glass coverslips. Following stimulation with IL-1β (25 ng/ml) for 30 min, the cells were washed twice with ice-cold PBS and fixed in 4% (w/v) paraformaldehyde. The cells were then quenched in 150 mM ammonium chloride, permeabilized in 0.1% (v/v) Triton X-100 in PBS, and blocked for 30 min in 5% (v/v) FBS in PBS. The cells were stained with rabbit anti-FLAG (Sigma, 1:300) and mouse anti-p65 antibody (Santa Cruz, 1:50) for 1 h, followed by incubation with goat anti-rabbit IgG Alexa Fluor 488 and goat anti-mouse IgG Alexa Fluor 568 secondary antibodies (Invitrogen). Coverslips were mounted in Mowiol 4-88 (Calbiochem) containing DAPI (4′,6-diamidino-2-phenylindole). Images were taken on a LSM 510 META confocal laser scanning microscope (Zeiss) using the LSM image browser software (Zeiss).

### Statistical Analysis

Statistical significance was determined using an unpaired Student's *t*-test with Welch's correction where appropriate.

## Data Availability Statement

The datasets generated for this study are available on request to the corresponding author.

## Author Contributions

IG planned and performed experiments, carried out data analysis, and prepared and edited the manuscript. CM planned and performed experiments, aided in data analysis, and wrote the manuscript.

### Conflict of Interest

The authors declare that the research was conducted in the absence of any commercial or financial relationships that could be construed as a potential conflict of interest.
