# Supplementary material for: Cullin-5 Adaptor SPSB1 Controls NF-κB Activation Downstream of Multiple Signaling Pathways
Source: Front Immunol. 2020 Jan 21;10:3121. doi: 10.3389/fimmu.2019.03121 (PMC6985365; doi:10.3389/fimmu.2019.03121)
Supplement: Supplementary file 1 [file Data_Sheet_1.PDF]

Cullin-5 adaptor SPSB1 controls NF-κB activation downstream of multiple signalling pathways

Georgana and Maluquer de Motes

Supplementary Material

Figure S1

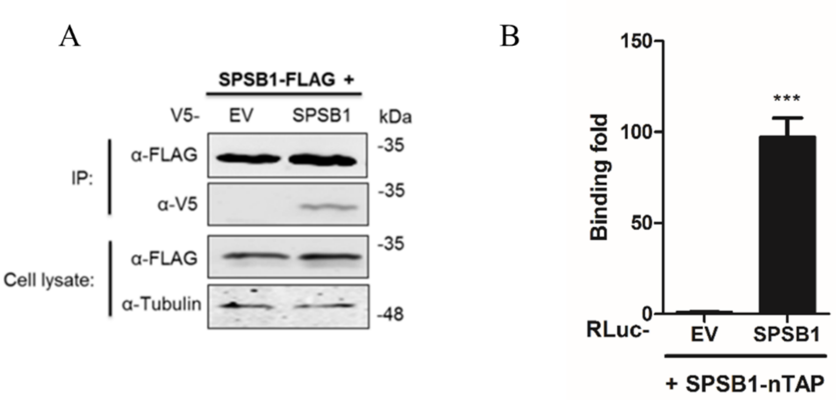

Figure S1. SPSB1 self-associates. (A) HEK293T cells were co-transfected with V5-tagged SPSB1, or an empty vector (EV) and FLAG-tagged SPSB1. The cells were lysed in IP buffer and subjected to FLAG IP. The IP and cell lysates were loaded onto an SDS-PAGE gel and immunoblotted against V5, FLAG and α-tubulin. (B) HEK293T cells were co-transfected in triplicates with RLuc-tagged SPSB1, or RLuc-only (EV) as a control and SPSB1-nTAP. Cells were lysed in IP buffer supplemented with protease inhibitors and lysates subjected to AP using streptavidin beads. The beads were eluted with biotin and the luciferase activity was measured both in lysates and eluates. The binding fold was calculated as an eluate/lysate ratio and this was normalised to the EV control. The experiments have been repeated three times independently. Means and standard deviations are shown and statistical significance was determined by using an unpaired Student's *t*-test (***$p \leq 0.001$).

Figure S2

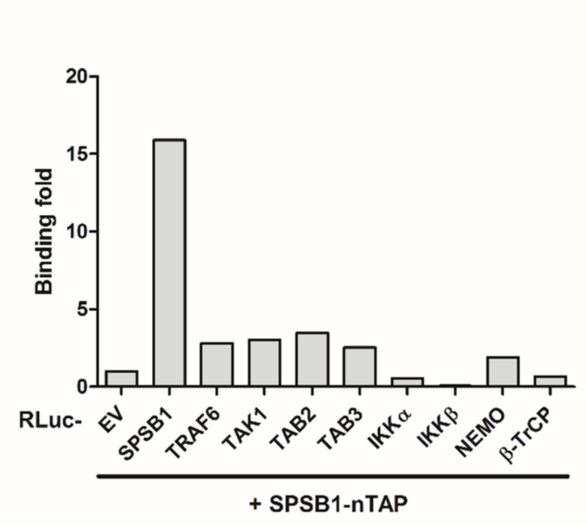

Figure S2. SPSB1 does not associate with any of the NF-κB components tested in LUMIER screens. HEK293T cells were co-transfected with SPSB1-nTAP and different components of the NF-κB pathway fused to RLuc, or an Rluc-only (EV) control. SPSB1 self-association was used a positive control. Cells were treated as in Fig. S1 and the binding fold was calculated as an eluate/lysate ratio and this was normalised to the EV control. Data are representative of 2 experiments showing similar results.

Figure S3

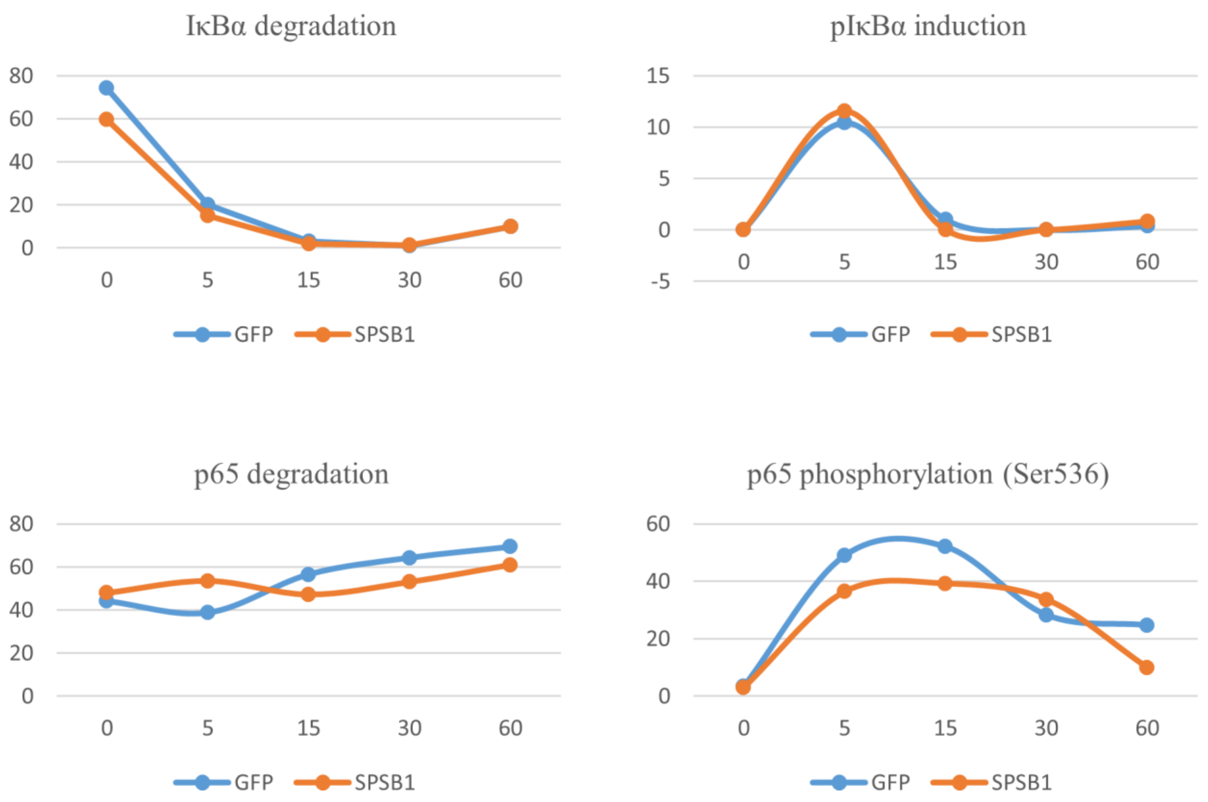

Figure S3. Densitometric analysis of the phosphorylation and degradation of IκBα and p65 in GFP- or SPSB1-expressing A549 cells. Immunoblotting results from Fig. 6 were quantitated (arbitrary units) and plotted over time.

Table S1. RNAi sequences employed for the specific knock-down of CRL5 substrate adaptor molecules.

Gene Symbol	Accession number	Duplex Number	Sequence
FBXW11	NM_033645	D-003490-02	GCACAUUGGUGGAACAUUC
FBXW11	NM_033645	D-003490-03	GUUAGUGGAUCAUCAGAUA
FBXW11	NM_033645	D-003490-04	GAGCAAGGCUUAGAUCACA
FBXW11	NM_033645	D-003490-17	AGAAGUAAAUCGACCGUCA
ASB1	NM_001040445	D-017197-01	GAAGAAAAGUGGACCCUGA
ASB1	NM_001040445	D-017197-02	GAGCCAACCUGAAUCUAGU
ASB1	NM_001040445	D-017197-03	GCAAACACCGGCUUCAUCU
ASB1	NM_001040445	D-017197-04	GCGCGAACCCUGACUUCAA
ASB2	NM_016150	D-009575-01	GAAAGGCCAUUGGGAAAUA
ASB2	NM_016150	D-009575-02	GCACGAGGCCGCAUACUAU
ASB2	NM_016150	D-009575-03	GAACAUCGACGCCUAUAUC
ASB2	NM_016150	D-009575-04	UUAGAUACCUGAAAUACGA
ASB3	NM_016115	D-017457-01	GGACAAAGCUACACCCUUG
ASB3	NM_016115	D-017457-02	GAAAGCUUGAGCAUACUUA
ASB3	NM_016115	D-017457-03	GAACGUCUACGGUCUGACA
ASB3	NM_016115	D-017457-04	CUUUGGAGUUUACUAAUUG
ASB4	NM_016116	D-013339-01	CUACAAAGCCGAAGUCAAU
ASB4	NM_016116	D-013339-02	CCAAGUUAGUUAAGAGAAA
ASB4	NM_016116	D-013339-03	CAAGGUUACUGGUUGCCUA
ASB4	NM_016116	D-013339-04	CCACAAUGCUACAAUCAAC
ASB5	NM_080874	D-017458-02	GGAUAUUGCUUCAACAUGA
ASB5	NM_080874	D-017458-03	GCCGUUUGCUCAACAAUUA
ASB5	NM_080874	D-017458-04	CAAGGAAGCGGCAAGGAUA
ASB5	NM_080874	D-017458-17	UGUAAUGGAUAACGUGCAU
ASB6	NM_017873	D-013355-01	GAACAUUCGUCUCUUACUG
ASB6	NM_017873	D-013355-02	GGAGGAUCAUCUUCGAGUA
ASB6	NM_017873	D-013355-03	GCAACGCCCUGCUCAAGAU
ASB6	NM_017873	D-013355-04	CUGAAGAGCUUUAAACUGC
ASB7	NM_024708	D-012930-01	GUGCACGGAUGUUAUAUAA
ASB7	NM_024708	D-012930-02	GGAGCGACAUCAUUAAUGC
ASB7	NM_024708	D-012930-03	GCGAUACGCCGUGAUCAAA
ASB7	NM_024708	D-012930-04	CGAACACACGGAACUAUGA
ASB8	NM_024095	D-014301-01	GGGCAGCCUUUAAGAACAA
ASB8	NM_024095	D-014301-02	GCGCUUAAUCCGAACAAUU
ASB8	NM_024095	D-014301-03	GCGCAGAGGUCAGAGUCAU
ASB8	NM_024095	D-014301-04	CUGGAUGGGUAUAACCGAA
ASB9	NM_024087	D-014295-01	GCAUCAGGCUUCUUUCAAA
ASB9	NM_024087	D-014295-02	GCAUGGAGCUCAGGUGAAU
ASB9	NM_024087	D-014295-03	GCACGGAGCCAGCGUUCAA
ASB9	NM_024087	D-014295-04	GAUGGGCGAUGCUGUGUCU
ASB10	NM_080871	D-007725-01	CAACAUCGCUGACCAGGAU

ASB10	NM_080871	D-007725-02	CAACAUCCGUGCUCUGAGA
ASB10	NM_080871	D-007725-04	GAGUUGGUCUCCAGAAGAG
ASB10	NM_080871	D-007725-17	GGUCUCUGACAUACGAAGA
ASB11	NM_001012428	D-013267-01	GAGCUAACCUGAAGCGUAG
ASB11	NM_001012428	D-013267-03	GCUCGGAACUCCCCUAUAU
ASB11	NM_001012428	D-013267-04	CAGAGCCACUCGAACGAUU
ASB11	NM_001012428	D-013267-18	CCGGAAGUGUCUCGGUCGA
ASB12	NM_130388	D-013180-01	GAAAUCUGCCUCCAUCAUA
ASB12	NM_130388	D-013180-02	GAAGCAGGCUCUCAAUCAA
ASB12	NM_130388	D-013180-03	GAGUAUAUCCAGCUGUUAA
ASB12	NM_130388	D-013180-04	CGUCAAAGCUAAACUACCA
ASB13	NM_024701	D-012929-01	GGAAGAAGCCGUCUGACUA
ASB13	NM_024701	D-012929-02	CCAAGUGCUUCGAGUACUA
ASB13	NM_024701	D-012929-03	GAGAAGAUUGCCAAGUUAA
ASB13	NM_024701	D-012929-04	CGAGAUGCUUAUCGAGUUU
ASB14	NM_130387	D-013205-01	GUUGAAGGCUGGACAUCUA
ASB14	NM_130387	D-013205-03	GAUACUAAGUUCUGUGAAG
ASB14	NM_130387	D-013205-04	CUGCAGAGUUAACCCUUUA
ASB14	NM_130387	D-013205-17	CCGCCUAAAGAUCCGGAAA
ASB15	NM_080928	D-031158-01	CGACUCUGAUCAAACAUAA
ASB15	NM_080928	D-031158-02	GAUAUUCAGCUAAGUAUUC
ASB15	NM_080928	D-031158-04	GUCAAGCGAUGGUCAGCAA
ASB15	NM_080928	D-031158-17	AGAGGAAGACUGCGCUGUA
ASB16	NM_080863	D-013061-01	CCGAGCUGCUGCUGCGUUA
ASB16	NM_080863	D-013061-02	AGACCCAGCUGUCCACCAA
ASB16	NM_080863	D-013061-03	AGACUGUGCUCGACACCUG
ASB16	NM_080863	D-013061-04	UCGCCGCACUGCUGGACUA
ASB17	NM_080868	D-015284-01	GAACUUUCACACCAGUAUA
ASB17	NM_080868	D-015284-02	GGAUAUCACUGUUACGAAC
ASB17	NM_080868	D-015284-03	AAUGGUUGAUCGUGAAUUG
ASB17	NM_080868	D-015284-04	UCACAGAUUACAUUGCAUU
ASB18	NM_212556	D-032545-02	CAAUAAGGAUGAGAUGGAA
ASB18	NM_212556	D-032545-03	GAAAUCUCCAGCCACGUUU
ASB18	NM_212556	D-032545-04	GAAGACCUGUGCAUCUGUC
ASB18	NM_212556	D-032545-17	CGGAUUACCUUCCCGACUA
CISH	NM_145071	D-017381-01	GACAACACCUGCAGAAGAU
CISH	NM_145071	D-017381-02	GAAGGCACGUUCUUAGUAC
CISH	NM_145071	D-017381-03	CCACUGCUGUACACCUAAA
CISH	NM_145071	D-017381-04	CCGACUACCUCCGACAGUA
RAB40A	NM_080879	D-008924-01	GCAAACCGCUGGUCUUUCG
RAB40A	NM_080879	D-008924-02	GGUAUGGAUCGAUGGAUUA
RAB40A	NM_080879	D-008924-03	CGACCAGGCCUAUGACUUC
RAB40A	NM_080879	D-008924-05	GGGUAUGGAUCGAUGGAUU
RAB40B	NM_006822	D-008353-01	CGGCAUUGAUCGAUGGAUU
RAB40B	NM_006822	D-008353-02	CGGUGCACCUGGUGGACAA
RAB40B	NM_006822	D-008353-03	CGGCGGGCAUCGACUACAA

RAB40B	NM_006822	D-008353-04	GGUCUAUGACAUUGCGAAC
RAB40C	NM_021168	D-010368-01	GCAGUCCGGUGAAGAGCUA
RAB40C	NM_021168	D-010368-02	CAGAACUGCUCGCGGAGUA
RAB40C	NM_021168	D-010368-03	GUAACGGGAUCGACUACAA
RAB40C	NM_021168	D-010368-04	GCGCGUACGCAGAGAAGAA
SOCS1	NM_003745	D-011511-07	GCAUCCGCGUGCACUUUCA
SOCS1	NM_003745	D-011511-08	GGUGGCAGCCGACAAUGCA
SOCS1	NM_003745	D-011511-09	GGACGCCUGCGGAUUCUAC
SOCS1	NM_003745	D-011511-23	UGUUAUUACUUGCCUGGAA
SOCS2	NM_003877	D-017604-01	GAAGGAACUUUCUUGAUUA
SOCS2	NM_003877	D-017604-02	GAAGUAUGACUGUUAAUGA
SOCS2	NM_003877	D-017604-03	GCAUUCAGACUACCUACUA
SOCS2	NM_003877	D-017604-04	UCAUAUGUGUCAAAUCCAA
SOCS3	NM_003955	D-004299-06	CCUAUUACAUCUACUCCGG
SOCS3	NM_003955	D-004299-08	UCAAGACCUUCAGCUCCAA
SOCS3	NM_003955	D-004299-21	CAGCAUCUCUGUCGGAAGA
SOCS3	NM_003955	D-004299-22	GCGAUGGAAUUACCUGGAA
SOCS4	NM_080867	D-009037-02	GCACAGCUGUUCAUCCAUU
SOCS4	NM_080867	D-009037-04	GAACUCAUGUUAGAUAAGU
SOCS4	NM_080867	D-009037-05	ACACGCAGAUUGAUUAUGU
SOCS4	NM_080867	D-009037-06	GCGCCUGUAUGUUCUUUGA
SOCS5	NM_014011	D-017374-01	CAACAAAGCAGUCCCUUAA
SOCS5	NM_014011	D-017374-02	AGACAGAGGUUGCAGGAUA
SOCS5	NM_014011	D-017374-03	GGAGGAAGCCGUAGUGAAA
SOCS5	NM_014011	D-017374-04	GAGAGAAGGCGGCUUAGUA
SOCS6	NM_004232	D-017375-01	GAGGAGACCUGCAUCAAGA
SOCS6	NM_004232	D-017375-03	GAACAUGUGCCUGUCGUUA
SOCS6	NM_004232	D-017375-04	GUCCAUAGUUGAUCUAAUU
SOCS6	NM_004232	D-017375-17	GAUAUGGCCAGCUGCGAUA
SOCS7	NM_014598	D-027197-01	GGAGAAGUGUGGUUGGUAU
SOCS7	NM_014598	D-027197-02	AAACCCAAGUUGACAAGAA
SOCS7	NM_014598	D-027197-03	UCCGAAAGUUCUACUACUA
SOCS7	NM_014598	D-027197-04	AAGCGCAGCUCAUUUCCAA
SPSB1	NM_025106	D-015262-01	GGACGACAAGCUCAUCUUU
SPSB1	NM_025106	D-015262-02	GAACAACAACGACCGAUCG
SPSB1	NM_025106	D-015262-03	ACUCUGAGCUUCAUUGUGG
SPSB1	NM_025106	D-015262-04	ACACAACCCUCGUGGGGAA
SPSB2	NM_032641	D-014947-02	GAACUCUGGGCUACGCUAU
SPSB2	NM_032641	D-014947-03	GACCCUCUAUCCGGCAGUA
SPSB2	NM_032641	D-014947-04	CUACCUGGGCGAAAGGAGA
SPSB2	NM_032641	D-014947-17	UGACAGUCUGCUACGCAAA
SPSB3	NM_080861	D-017713-01	GGUCUGGGAUGACUUAAAU
SPSB3	NM_080861	D-017713-02	GAUGACCUCUCCCGUCUAC
SPSB3	NM_080861	D-017713-03	ACAAAUACCGCCACACGUU
SPSB3	NM_080861	D-017713-04	GCGGAGAGGAAGACGAGUA
SPSB4	NM_080862	D-015283-01	GCAAGAAGCUGUACCCGGU

SPSB4	NM_080862	D-015283-02	ACACUCAGCUUCAUCGUGG
SPSB4	NM_080862	D-015283-03	AAAACUAUCUGCAGUACCA
SPSB4	NM_080862	D-015283-04	GCACCCACGCUGUAGUUGG
WSB1	NM_134265	D-013015-01	UAUGGGACCUGAAAGAUGA
WSB1	NM_134265	D-013015-02	GGAAACAUGAUGAAAGUAU
WSB1	NM_134265	D-013015-03	GAAGUGGUCAGAGAUUUAA
WSB1	NM_134265	D-013015-04	GAAAACUCCUCCUUAACUU
WSB2	NM_018639	D-017223-01	CAUGAGGUCCUACACGUUA
WSB2	NM_018639	D-017223-02	CGAUGUCUCUUGCCUGGUU
WSB2	NM_018639	D-017223-03	CAAGAUGUCGUGAGAGAUC
WSB2	NM_018639	D-017223-04	CCGGAAAGCCCUUCGAAGU
